# Cure of Tooth-Ache—Destruction of Living Nerve, and Removal of the Tenderness of the Bony Cavity of a Decayed Tooth, without Pain in Most Cases, and with Slight Pain in Any

**Published:** 1841

**Authors:** Solyman Brown


*Cure of Tooth-Ache,—destruction of living nerve, and removal of the tenderness of the bony cavity of a decayed tooth, without pain in most cases, and with slight pain in any.*—By S. Brown M. D.

Of all the remedies for the above diseases, which have hitherto been generally made known to the profession in this counrty, arsenic is unquestionably the most prompt, safe and effectual, when properly applied.

1. To a hollow tooth the bony parietes of which are too sensitive to be prepared for stopping, the dry arsenic may be applied in a small quantity on a lock of moist cotton of sufficient size to fill half the cavity when moderately compressed. Over this, wax, tinfoil, or dry cotton, should be placed in such a manner as to prevent the escape of the medicine into the mouth. A very minute portion of the arsenic is sufficient, provided it can be completely confined to the cavity of the tooth.

2. To a carious opening which is the immediate cause of tooth-ache, as well as to one in which a living nerve must be destroyed in order to prepare the tooth for stopping, the arsenic should be applied on the extremity of a lock of cotton, steeped in creosote instead of water. The effect of the creosote is to allay the pain which the arsenic alone would produce when acting on the living nerve. In the former case (No. 1.) no creosote is necessary, because no nerve is present. In all these cases it will be well to apply the remedy in the early part of the day, in order that the patient may be able to use proper caution in preventing the removal of the cotton, and also be careful to keep the mouth free from any portion of the medicine which may make its escape. Excoriations of the lips, tongue and gums, may be the unpleasant consequences of applying the remedy in the after part of the day, in case, during sleep, the cotton should be displaced. It need scarcely be remarked that a powerful and dangerous remedy like this, should be used with the utmost care.

